# Dietary phosphorus and calcium in feed affects miRNA profiles and their mRNA targets in jejunum of two strains of laying hens

**DOI:** 10.1038/s41598-021-92932-3

**Published:** 2021-06-29

**Authors:** Muhammad Arsalan Iqbal, Asghar Ali, Frieder Hadlich, Michael Oster, Henry Reyer, Nares Trakooljul, Vera Sommerfeld, Markus Rodehutscord, Klaus Wimmers, Siriluck Ponsuksili

**Affiliations:** 1 Leibniz Institute for Farm Animal Biology, Institute of Genome Biology, Wilhelm-Stahl-Allee 2, 18196 Dummerstorf, Germany; 2 Institute of Animal Science, University of Hohenheim, 70599 Stuttgart, Germany; 3 Faculty of Agricultural and Environmental Sciences, University Rostock, 18059 Rostock, Germany

**Keywords:** Biotechnology, Genetics, Gastroenterology

## Abstract

Phosphorus (P) and calcium (Ca) are critical for egg production in laying hens. Most of P in plant-based poultry diet is bound as phytic acid and needs to be hydrolysed before absorption. To increase P bioavailability, exogenous phytases or bioavailable rock phosphate is added in feed. There is growing evidence of the importance of miRNAs as the epicentre of intestinal homeostasis and functional properties. Therefore, we demonstrated the expression of miRNA profiles and the corresponding target genes due to the different levels of P (recommended vs. 20% reduction) and/or Ca (recommended vs. 15% reduction) in feed. Jejunal miRNA profiles of Lohmann Selected Leghorn (LSL) and Lohmann Brown (LB) laying hens strains were used (n = 80). A total of 34 and 76 miRNAs were differentially expressed (DE) in the different diet groups within LSL and LB strains respectively. In LSL, the DE miRNAs and their targets were involved in calcium signaling pathway, inositol phosphate metabolism, and mitochondrial dysfunction. Similarly, in LB miRNAs targets were enriched in metabolic pathways such as glutathione metabolism, phosphonate metabolism and vitamin B6 metabolism. Our results suggest that both strains employ different intrinsic strategies to cope with modulated P and Ca supply and maintain mineral homeostasis.

## Introduction

An increase in food production is critical to meet the challenge of increasing world's population. Eggs are one of the most affordable source of proteins and the number of laying flocks is rapidly increasing all over the world^1^. One of the major factors impacting the egg production is egg shell quality which also poses huge economic losses to the industry. Calcium (Ca) is one of the most important minerals which is required for rapid mineralization during egg shell formation^2^. Ca is also critical in formation and strength of skeleton, muscle conduction and blood coagulation^3^. In case of insufficient dietary Ca supply or improper Ca absorption from the gastrointestinal tract in laying hens, Ca is mobilized from bones^4,5^. Therefore, a disturbance in Ca homeostasis can deteriorate both egg quality and health of the bird. Intestinal Ca absorption, its storage, mobilisation and retention is strictly regulated together with phosphorus (P) and depending on Ca to P ratio by endocrine factors including parathyroid hormones and levels of vitamin D_3_^6,7^. Phosphorus is an essential nutrient in all organisms and a critical component of membrane phospholipids, bones and genetic material, and is also required for proper utilization of Ca^8–10^. Deficiency of P or Ca can lead to reduced appetite, abnormal skeleton development, reduced growth in young birds, and osteoporosis and weight loss in older birds^11,12^. Several factors affect the absorption and utilization of P and Ca in laying hens including impaired P to Ca ratio, their interaction with other nutrients, the genetic background, microbiota, and environmental conditions^13^.

Both P and Ca are absorbed primarily in duodenum and jejunum^14,15^, but the extent of absorption depends on their binding form in the feed. Most of P in plant-based poultry feed is bound as phytic acid (*myo*-inositol 1, 2, 3, 4, 5, 6-hexakis dihydrogen phosphate; InsP_6_) or its salt called phytate^16^. The stepwise breakdown of InsP_6_ in the gastrointestinal tract (GIT) by phytases and phosphatases yields bioavailable P and myo-inositol (MI) as end product^17^. Low bioavailability of P from plant-based feed led to the use of feed phosphate in poultry feed^18^. Feed phosphate is produced from rock phosphate, which is non-renewable and if used at current rate, the global P reserves will be depleted in 50–100 years^19,20^. Therefore, there is an urgent need to increase the bioavailability of P from plant-based feed. In addition, not only the limited resources of P but also its potential environmental impact due to excessive use in agricultural production require a system change towards more efficient use.

Nutrient absorption from the GIT is extensively regulated by a number of transcellular (active) and paracellular (passive) mechanisms, which include e.g. the vitamin D system. In fact, vitamin D_3_ plays an important role in the utilization of Ca and P and their absorption from the small intestine^21^. Calcitriol, or 1,25- dihydroxyvitamin D_3_ (1,25-(OH)_2_ D_3_), the active form of vitamin D_3_, stimulates both paracellular and transcellular absorption of calcium in gastrointestinal tract^22^. Other than vitamin D_3_, several transporters and molecular pathways are involved in absorption and retention of Ca and P. The proteins of Na/Pi-II family are located at apical surface of intestinal epithelial cells and mediate P absorption^23,24^. In chickens, the Na/Pi cotransporter NPT2a is expressed in kidneys and mediates reabsorption of P^25^. The expression of several genes, including the genes and molecular pathways involved in nutrients absorption in small intestine, is regulated by microRNAs. For instance, miR-101, miR-1253, miR-1224-5p, miR-1226-55p and miR-623 play a pivotal role in regulation of growth of specific microbiota in gut intestine^26^.

MicroRNAs (miRNAs) are highly conserved short non-coding RNAs which regulate gene expression by degradation or translational inhibition of their target mRNAs^27^. MiRNAs regulate several biological and cellular processes in a variety of tissues across different animal species^28–30^. Various networks of miRNAs were identified in intestinal epithelial cells, which indicates the potential role of miRNAs in regulating nutrient metabolism and intestinal secretions^31^. Several nutrimiromics studies have demonstrated that different minerals and vitamins can alter the expression of miRNAs^32^. We previously revealed molecular interactions occurring in the gut of Japanese quail which represented extremes for P utilization including miRNA-16-5p, miR-142b-5p, miR-148a-3p^33^. There is a strong possibility that miRNAs expressed in intestinal epithelial cells in laying hens are involved in regulating the phytate hydrolysis and other important processes related to mineral absorption and utilization in GIT.

In addition, previous studies revealed that the different strains of laying hens, Lohmann Brown (LB) and Lohmann Selected Leghorn (LSL), express similar performance on eggs production but exhibit pronounced differences in body weight, immunity, bone metabolism and capacity of phytate degradation^34–38^. Therefore, both laying strains were used in this study to further determine strain-specificities in mineral homeostasis. However, the current knowledge on the molecular regulation of Ca–P homeostasis, especially in relation to gene expression including the regulation by miRNAs in gut, is still limited.

Using a nutrimiromics approach we investigated the impact of dietary mineral supplies on the expression of miRNAs in jejunum retrieved from LB and LSL laying hen strains in order to elucidate the interaction between feed ingredients and miRNA networks in small intestine. The LB and LSL hens were fed with different levels of Ca and P in their feed. The aim was to identify miRNAs and downstream pathways involved in mineral absorption and utilization, and metabolism in jejunum tissue.

## Results

### Mapping of miRNAs

MiRNA sequencing of 80 libraries generated 578,617,302 raw reads. The 80 miRNA libraries were equally distributed between LSL and LB strains and each strain had 40 sequencing samples. After adaptor trimming, size selection and quality filtering, reads with length of 18–33 nucleotides and a phred score > 20 were retained for mapping (Table S1). 523,291,583 out of 532,225,124 reads for LSL strain and 454,205,567 out of 463,921,782 reads for LB strain were uniquely mapped to *Gallus gallus* reference genome (GRCg6a). The overall percentage of mapping reads was 98.31% in LSL strain and 97.75% in LB strain.

### Differential expression of miRNAs

**Figure 1:**
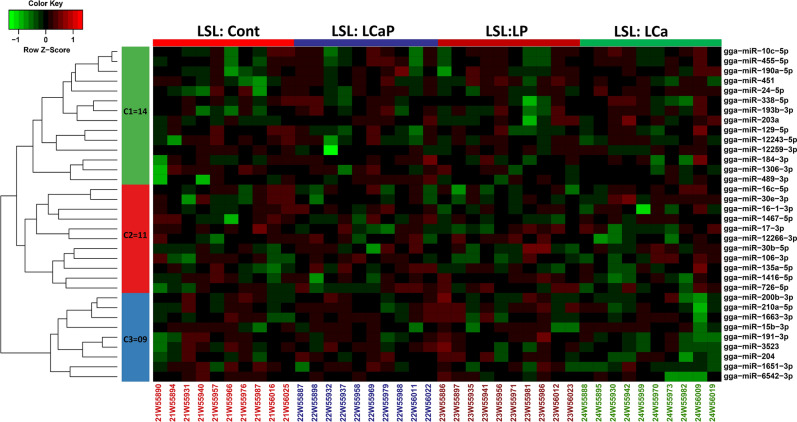
Expression profile of known miRNAs between diet groups within LSL strain. Heatmap of miRNAs expression profiles was generated in the R Programming environment (version 3.4.3) (https://www.R-project.org/) by using the hierarchical clustering method of heatmap.2 function of gPlots (version 3.0.1) (https://CRAN.R-project.org/package=gplots). MiRNAs were assigned to three clusters based on their co-expression with cut-off criteria of variance-stabilizing transformations ≥ 2, |logFC ≥ 1|. A total of 34 differentially expressed known miRNAs were distributed in three clusters including 14, 11 and 9 miRNAs in cluster one, two and three respectively. In color key: green color represents downregulation, red color represents upregulation and black color represents no change in expression.

**Figure 2:**
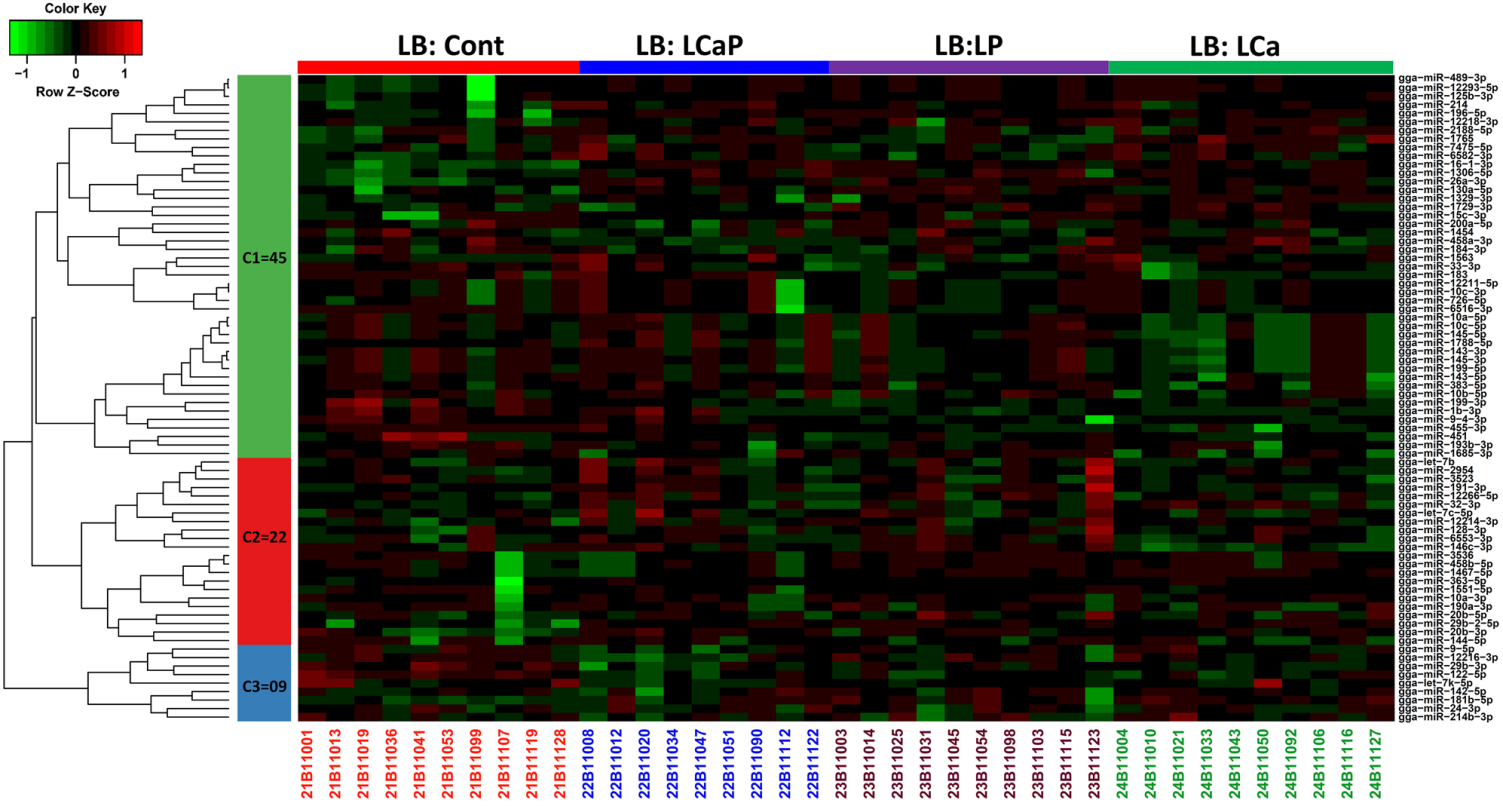
Expression profile of known miRNAs between diet groups within LB strain. Heatmap of miRNA expression profiles was generated in the R Programming environment (version 3.4.3) (https://www.R-project.org/) by using the hierarchical clustering method of heatmap.2 function of gPlots (version 3.0.1) (https://CRAN.R-project.org/package=gplots). MiRNAs were assigned to three clusters based on their co-expression with cut-off criteria of variance-stabilizing transformations ≥ 2, |logFC ≥ 1|. A total of 76 differentially expressed known miRNAs were distributed in three clusters including 45, 22 and 9 miRNAs in cluster one, two and three respectively. In color key: green color represents downregulation, red color represents upregulation and black color represents no change in expression.

Based on data pre-processing and filtration, overall 576 miRNAs were selected for differential expression analysis. First, the differential expression analysis of miRNAs was performed between LSL and LB strains within each diet group. The number of differentially expressed (DE) miRNAs between the two strains is included in Table 1. For each diet group, the differentially expressed known miRNAs between strains ranged from 116 to 133 with P < 0.05 and 88 to 93 with threshold of 5% FDR (Table S2). Next, the differential expression analysis of miRNAs was performed between different diet groups within each strain of laying hens. Only the known miRNAs that were differentially expressed between diet groups within LSL and LB strains were considered and are listed in Table 2. A total of 34 and 76 known miRNAs were differentially expressed in the different diet groups within LSL and LB strains respectively (Table S3 and Table S4). The number of differentially expressed known miRNAs between different diet groups ranged from 2–10 in LSL strain and 7–21 in LB strain at a cut-off of *P* < 0.05. Heatmaps of differentially expressed known miRNAs among diet groups are shown in Fig. 1 (LSL) and Fig. 2 (LB). In total, 34 differentially expressed miRNAs were clustered in three groups including 14, 11 and 9 miRNAs in cluster one, two and three, respectively in LSL strain whereas in LB strain, 76 differentially expressed miRNAs were clustered in three groups including 45, 22 and 9 miRNAs in cluster one, two and three, respectively based on their co-expression.

**Table 1:** The number of differentially expressed (DE) miRNAs between strains (Lohmann Selected Leghorn (LSL) and Lohmann Brown (LB)) within diet groups at cut-offs of *P* < 0.05 and FDR < 0.05.

Diets groups	DE miRNAs (LSL and LB)	
	*P* < 0.05	FDR < 0.05
Cont	133	92
LCaP	119	93
LCa	120	92
LP	116	88

**Table 2:** The number of differentially expressed (DE) known miRNAs from comparisons between diet groups within strains (Lohmann Selected Leghorn (LSL) and Lohmann Brown (LB)) and their upregulated and downregulated target genes at cut-off of *P* < 0.05.

Diets	DE miRNA (LB)	DE mRNA targets (LB)		DE miRNA (LSL)	DE mRNA targets (LSL)	
	Up/down	Up	Down	Up/down	Up	Down
Cont *vs* LCaP	09	12	35	10	25	69
Cont *vs* LCa	07	11	1	02	10	04
Cont *vs* LP	16	25	49	08	113	75
LCaP *vs* LCa	12	97	33	02	05	07
LCaP *vs* LP	13	09	02	03	13	07
LCa *vs* LP	21	37	24	07	26	95

### Correlation network analysis of differentially expressed miRNAs and their target genes between different dietary groups within strains

**Figure 3:**
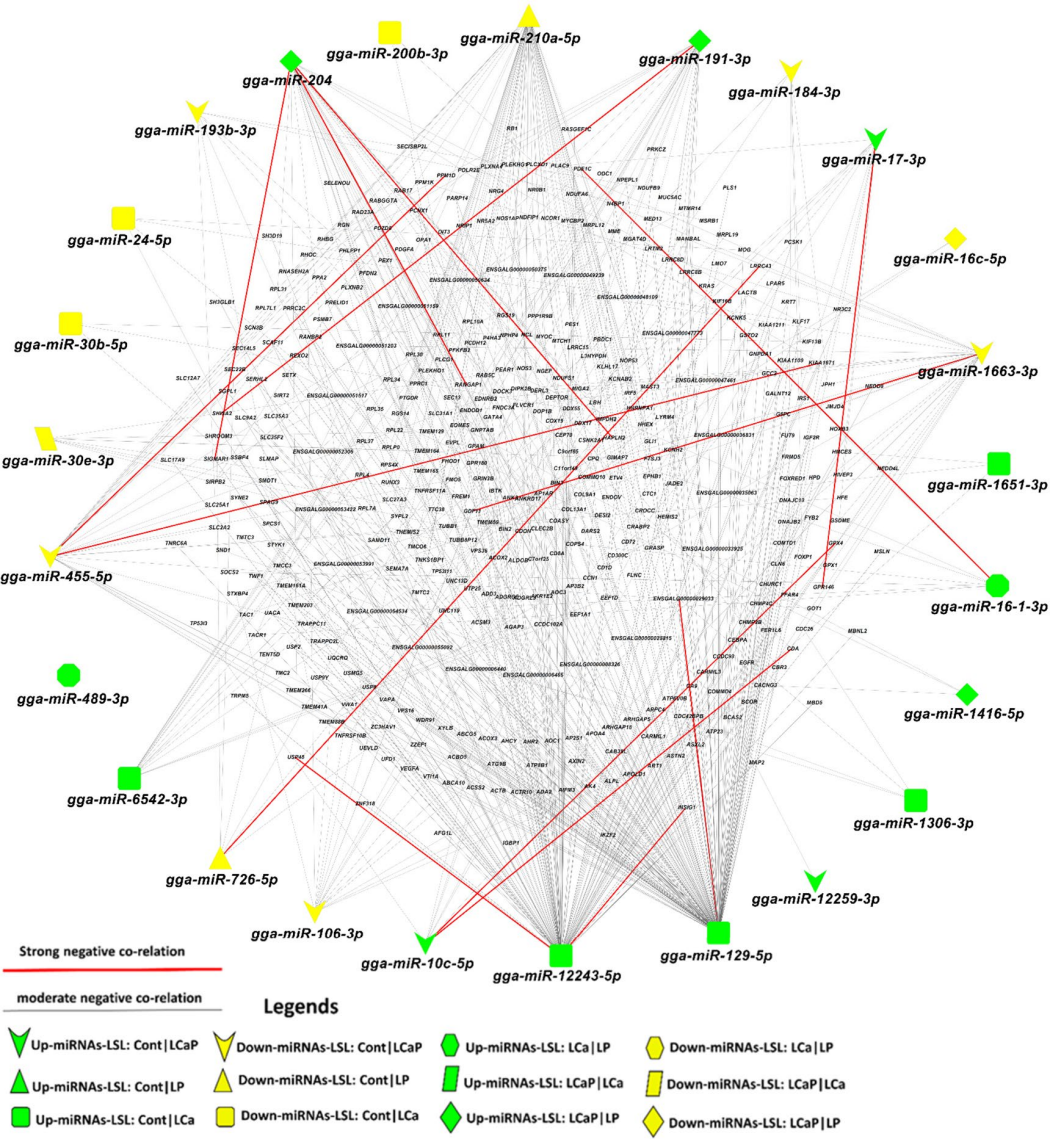
Correlation network analysis of differentially expressed (DE) known miRNAs and their DE target genes from pairwise comparisons between diet groups within LSL strain. Correlation network was generated using MetScape (version 3.1.3) plugin in Cytoscape (version 3.6.1) environment. The mRNAs with negative correlation with miRNAs were considered significant. Green color indicates upregulated miRNAs and yellow color indicates downregulated miRNAs. Red bold line indicates the strong negative correlation ranging from − 0.35 to − 0.60 and grey line indicates moderate negative correlation ranging from − 0.30 to − 0.35 according to their correlation coefficient score.

**Figure 4:**
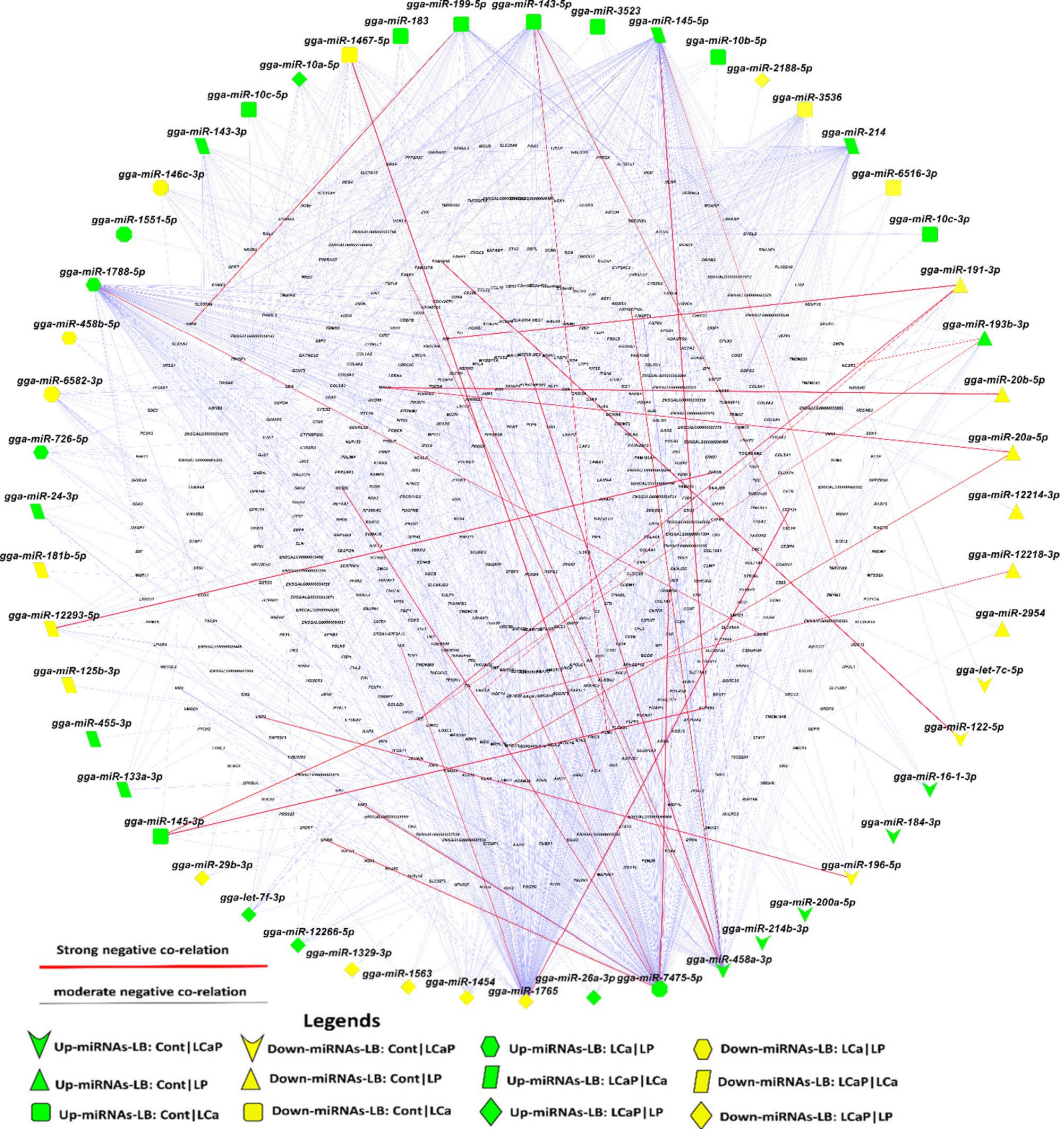
Correlation network analysis of differentially expressed (DE) known miRNAs and their DE target genes from pairwise comparisons between diet groups within LB strain. Correlation network was generated by using MetScape (version 3.1.3) plugin in Cytoscape (version 3.6.1) environment. The mRNAs with negative correlation with miRNAs were considered significant. Green color indicates the upregulated miRNAs and yellow color indicates downregulated miRNAs. Red bold line indicates the strong negative correlation ranging from − 0.35 to − 0.80 and blue line indicates moderate negative correlation ranging from − 0.30 to − 0.35 according to their correlation coefficient score.

Data of DE mRNAs/genes in jejunum from the same birds was obtained, which were analysed in a separate study with (accession number: E-MTAB-9109). Pearson correlation coefficients between DE miRNAs and DE genes were determined for all dietary combinations. DE genes that were negatively correlated with DE miRNAs were considered significant at threshold of 5% FDR. In different diet groups of the LSL strain, 25 out of 34 DE miRNAs were negatively correlated with 448 DE target genes which passed the significant threshold of FDR < 0.05, whereas 9 DE miRNAs showed no correlation with any DE genes (Fig. 3). Out of 448 DE target genes in LSL diet groups, 195 were upregulated and 253 were downregulated (Fig. 3). Similarly, in different diet groups of the LB strain, 52 out of 76 DE miRNAs were negatively correlated with 336 DE target genes which passed the significant threshold of FDR < 0.05, 24 DE miRNAs showed no correlations (Fig. 4). Out of 336 DE target genes in LB diet groups, 192 were upregulated and 144 were downregulated (Fig. 4). Moderate negative correlation ranged from − 0.30 to − 0.35 and strong negative correlation ranged from − 0.35 to − 0.60 in LSL strain, whereas in LB strain modest negative correlation ranged from − 0.30 to − 0.35 and strong negative correlation ranged from − 0.35 to − 0.80 with respect to their correlation coefficient score.

### Gene ontology enrichment analysis for target genes of DE miRNAs

**Figure 5:**
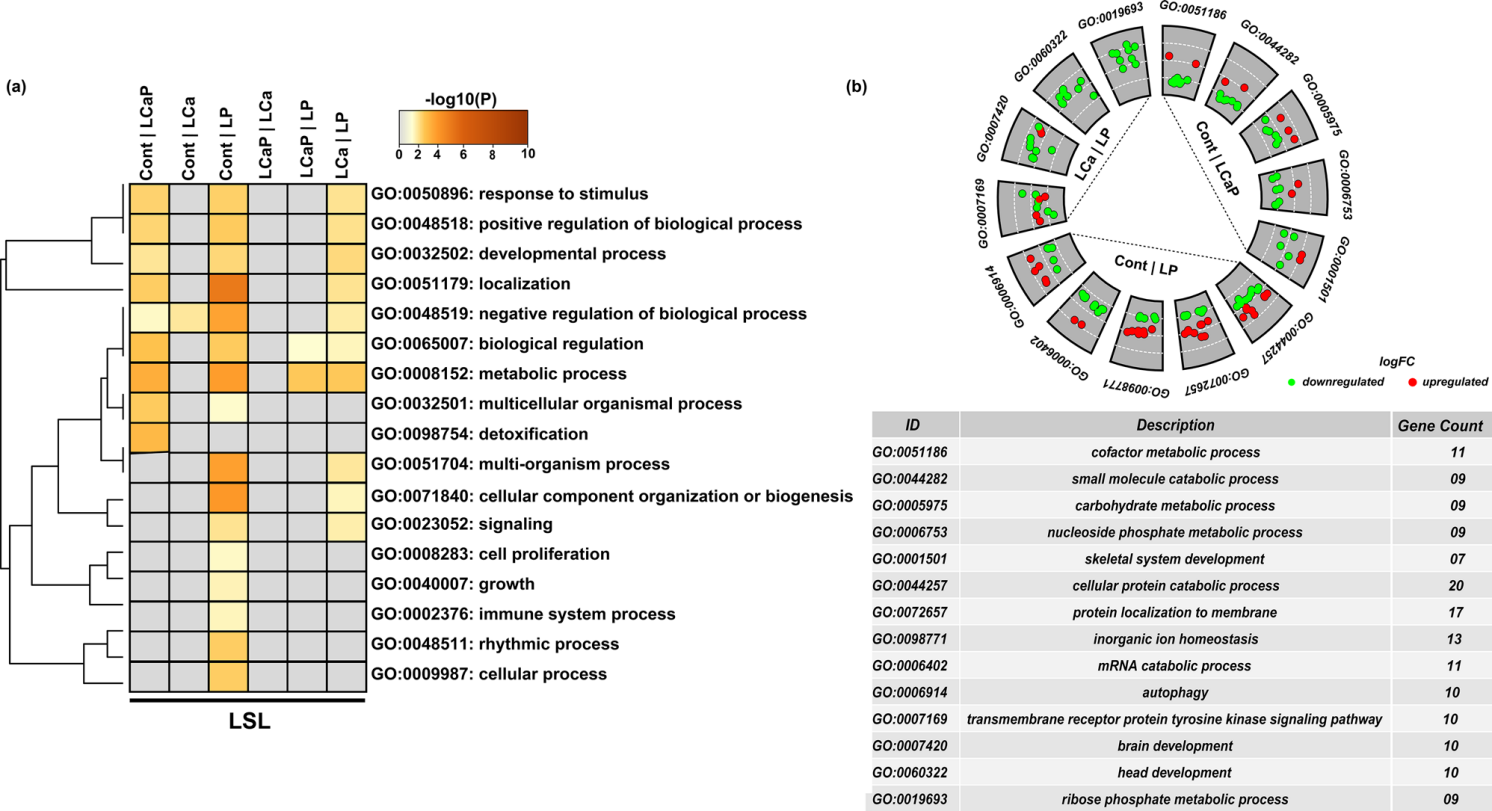
Gene ontology enrichment analysis of differentially expressed (DE) known miRNAs and their DE target genes from pairwise comparisons between diet groups within LSL strain. (**a**) Enrichment analysis of DE miRNAs and their target genes from different dietary comparisons was performed in Metascape. All statistically enriched biological processes with accumulative hypergeometric *P* values and enrichment score were identified. Significant biological terms were then hierarchically clustered into a tree based on Kappa-statistical similarities among their genes. Kappa score 0.3 was applied as threshold to cast the tree into biological term clusters. The heatmap cells are colored by their *P* values. Grey color indicates lack of enrichment and brown color indicates significant enrichment for that term in corresponding group gene list. (b) Go circular plot was generated by using Goplot (version.1.0.2) (https://CRAN.R-project.org/package=GOplot) within R programming environment (https://www.R-project.org/). Only the dietary comparison groups, which indicated the significant enrichment, were selected. Top five biological processes were selected from these enriched groups based on number of genes involved in a biological process. White dotted line indicates the partition of logFC from low to high level.

**Figure 6:**
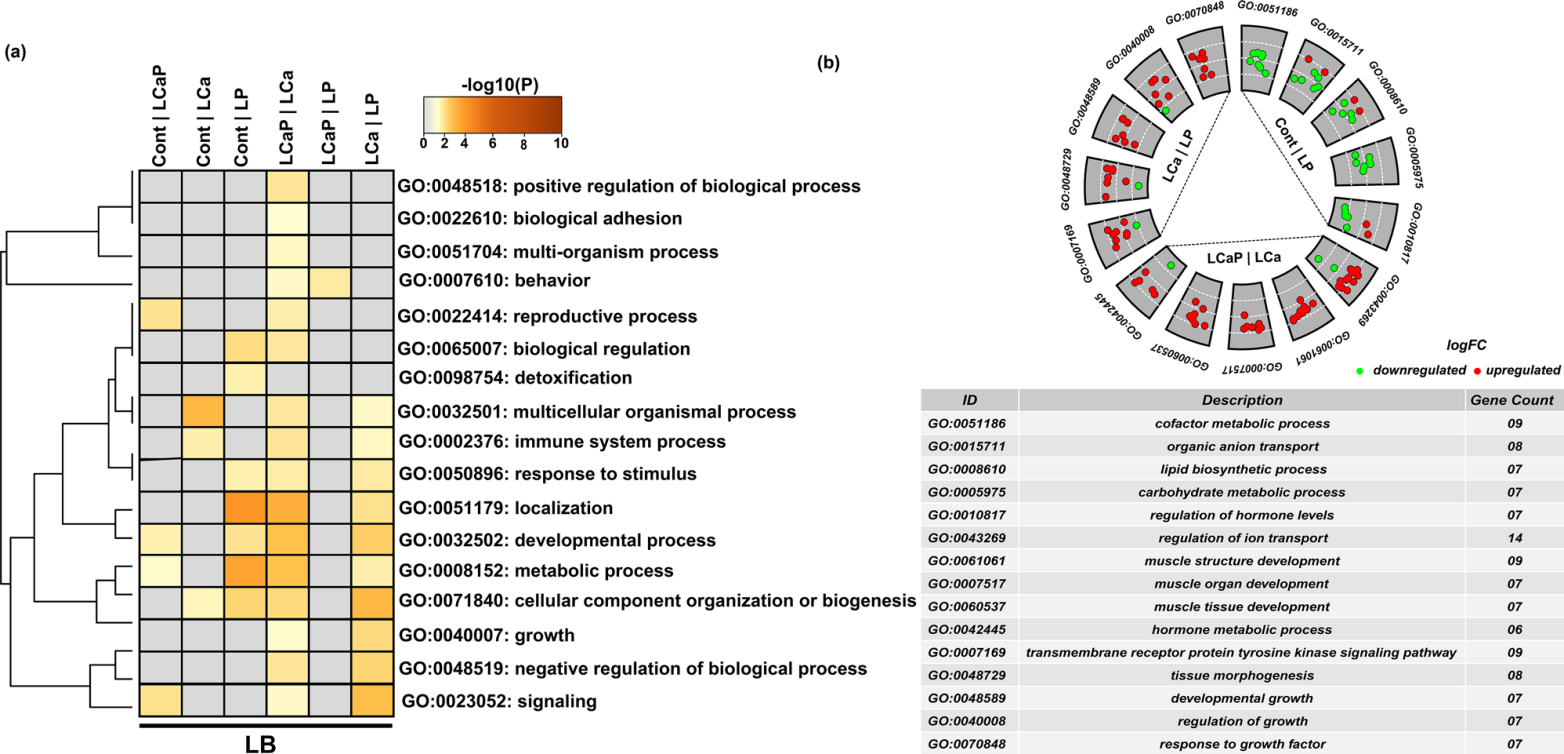
Gene ontology enrichment analysis of differentially expressed (DE) known miRNAs and their DE target genes from pairwise comparisons between diet groups within LB strain. (**a**) Enrichment analysis of DE miRNAs and their target genes from different dietary comparisons was performed in Metascape. All statistically enriched biological processes with accumulative hypergeometric *P* values and enrichment score were identified. Significant biological terms were then hierarchically clustered into a tree based on Kappa-statistical similarities among their genes. Kappa score 0.3 was applied as threshold to cast the tree into biological term clusters. The heatmap cells are colored by their *P* values. Grey color indicates lack of enrichment and brown color indicates significant enrichment for that term in corresponding group gene list. (**b**) Go circular plot was generated by using Goplot (version.1.0.2) (https://CRAN.R-project.org/package=GOplot) within R programming environment (https://www.R-project.org/). Only the dietary comparison groups, which indicated the significant enrichment, were selected. Top five biological processes were selected from these enriched groups on the basis of number of genes involved in a biological process. White dotted line indicates the partition of logFC from low to high level.

To investigate the function of DE target genes of DE miRNAs, gene ontology enrichment analysis was performed using Metascape for six possible comparisons between four diet groups within strains. DE target genes from Cont *vs* LCaP, Cont *vs* LP and LCa *vs* LP comparisons were significantly enriched in gene ontology top-level biological processes (parent biological process) such as response to stimulus, positive regulation of biological process, developmental process and metabolic process with respect to their P values (Fig. 5a). Whereas, DE target genes from other three comparisons including Cont *vs* LCa, LCaP *vs* LCa and LCaP *vs* LP were not significantly enriched in biological process (Fig. 5a). Similarly, in LB strain, DE target genes from only Cont *vs* LP, LCaP *vs* LCa and LCa *vs* LP comparisons were significantly enriched in gene ontology parent biological processes such as multicellular organismal process, immune system process, response to stimulus, localization, developmental process and metabolic process (Fig. 6a). Afterward, we selected the top five biological processes of only those comparisons which showed enrichment in LSL and LB strains.

In Cont *vs* LCaP comparison within LSL strain, 12 DE miRNAs with 94 DE target genes (25 upregulated and 69 downregulated in Cont group) were involved in cofactor metabolic process, small molecule catabolic process, carbohydrate metabolic process, nucleoside phosphate metabolic process, and skeletal system development. Interestingly, the downregulated genes showed more involvement in these biological processes (Fig. 5b). In Cont *vs* LP comparison within LSL strain, 8 DE miRNAs with 188 DE target genes (113 upregulated and 75 downregulated in Cont group) were involved in cellular protein catabolic process, autophagy, protein localization to membrane, inorganic ion homeostasis and genes catabolic process (Fig. 5b). In LCa *vs* LP comparison within LSL strain, 7 DE miRNAs with 121 DE target genes (26 upregulated and 95 downregulated in LCa group) were involved in transmembrane receptor protein tyrosine kinase signalling pathway, brain development, head development and ribose phosphate metabolic process. The downregulated genes showed more involvement in these biological processes (Fig. 5b).

In Cont *vs* LP comparison within LB strain, 16 DE miRNAs with 74 DE target genes (25 upregulated and 49 downregulated in Cont group) were significantly enriched in cofactor metabolic process, lipid biosynthetic process, carbohydrate metabolic process, organic anion transport and regulation of hormone levels. The downregulated genes showed more involvement in these biological processes (Fig. 6b). In LCaP *vs* LCa comparison within LB strain, 12 DE miRNAs with 130 DE target genes (97 upregulated and 33 downregulated in LCaP group) were involved in muscle structure development, muscle organ development, muscle tissue development, hormone metabolic process and regulation of ion transport. The upregulated genes showed more involvement in these biological processes (Fig. 6b). In LCa *vs* LP comparison within LB strain, 21 DE miRNAs with 61 DE target genes (37 upregulated and 24 downregulated in LCa group) were enriched in developmental growth, regulation of growth, tissue morphogenesis, transmembrane receptor protein tyrosine kinase signalling pathway and response to growth factor. The upregulated genes showed more involvement in these biological processes (Fig. 6b).

### KEGG pathway enrichment analysis of target genes of DE miRNAs

**Figure 7:**
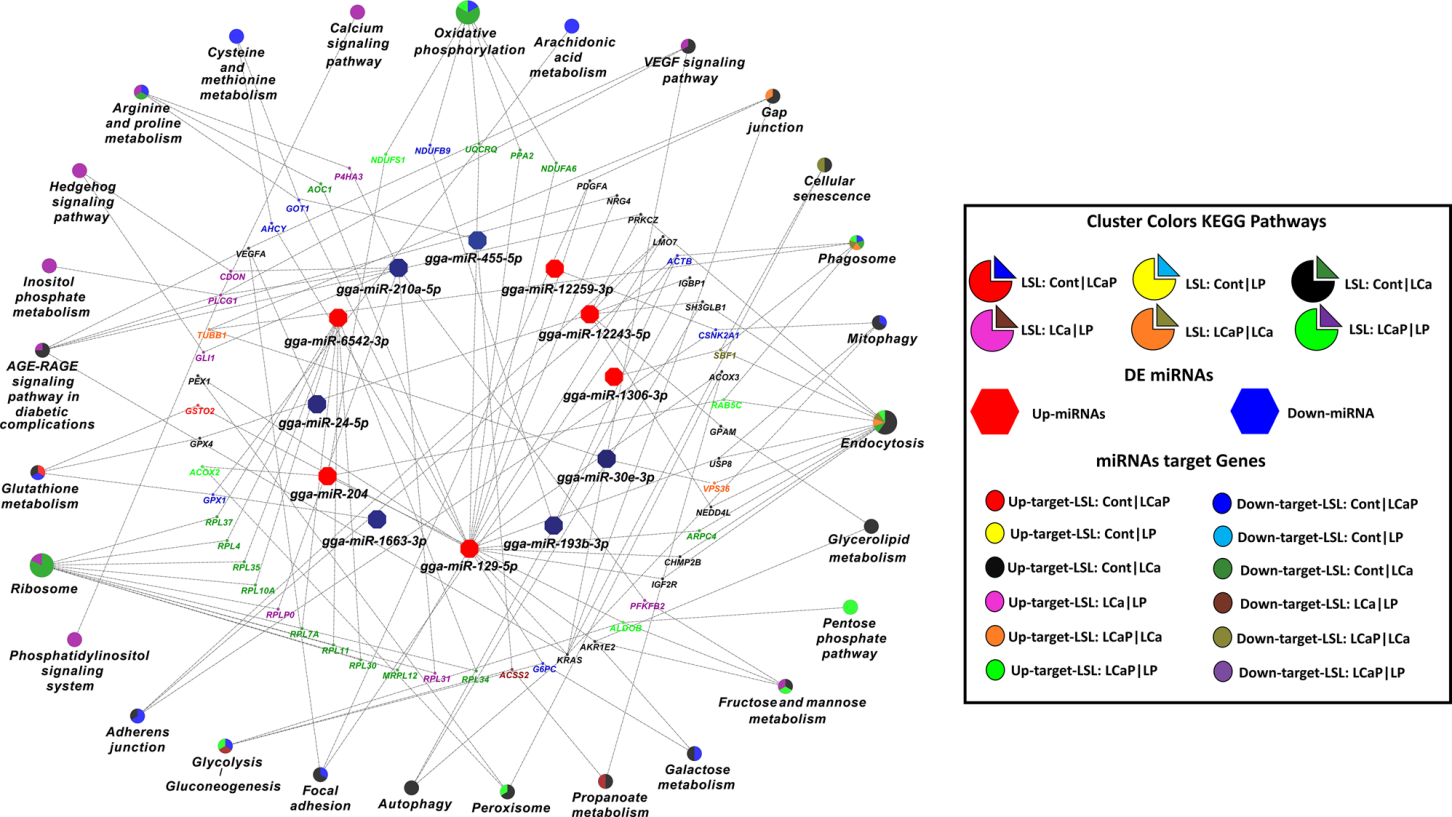
KEGG pathways enrichment analysis of differentially expressed (DE) known miRNAs and their DE target genes from pairwise comparisons between diet groups within LSL strain. KEGG pathways enrichment analysis was performed by ClueGO (version 2.5.1) and Cluepedia (version 1.5.7) plugin in Cytoscape (version.3.6.1) environment. ClueGO generated functionally clustered KEGG annotation network of DE miRNAs and their DE target genes. *P* value was calculated by right-sided hypergeometric tests and Benjamin–Hochberg adjustment was used for multiple test correction. KEGG pathways with a *P* < 0.05 were considered significant.

KEGG pathways enrichment analysis was performed for DE miRNAs and their DE target genes in all six dietary comparisons within LSL and LB strains. Initially, 34 DE miRNAs and 448 DE target genes (195 upregulated and 253 downregulated) from 6 possible comparisons within LSL strain were subjected to ClueGO and Cluepedia plugin in Cytoscape environment. 12 clusters were used for 6 comparisons, where one cluster of upregulated target genes and one cluster of downregulated target genes was used for each dietary comparison. Functionally annotated KEGG pathway networks generated by ClueGO showed that 12 out of 34 DE miRNAs (6 upregulated and 6 downregulated) and their target genes were involved in KEGG pathways. Moreover, DE miRNAs and their target genes from Cont *vs* LCaP, Cont *vs* LP and LCa *vs* LP comparisons within LSL strain were significantly enriched in KEGG pathways and the other three dietary comparisons (Cont *vs* LCa, LCaP *vs* LCa and LCaP *vs* LP) had no significant enrichment. In Cont *vs* LCaP comparison within LSL strain, miRNAs gga-miR-129-5p and gga-mir-12259-3p were downregulated and gga-miR-455-5p, gga-miR-193b-3p and gga-miR-1663-3p were upregulated in LCaP group. These DE miRNAs and their 94 target genes (25 upregulated and 69 downregulated in Cont group) were involved in phagosome, mitophagy, oxidative phosphorylation, glutathione metabolism, cysteine and methionine metabolism and galactose metabolism. The downregulated genes showed more involvement in these pathways (Fig. 7). In Cont *vs* LP comparison within LSL strain, gga-miR-129-5p, gga-miR-6542-3p and gga-miR-12243-5p were downregulated, and gga-miR-24-5p was upregulated in LP group. These DE miRNAs and their 188 DE target genes (113 upregulated and 75 downregulated in Cont group) were involved in ribosome, autophagy, endocytosis, mitophagy, focal adhesion, oxidative phosphorylation, glycerolipid metabolism and galactose metabolism. The upregulated genes showed more involvement in these pathways (Fig. 7). In LCa *vs* LP comparison within LSL strain, gga-miR-204 was upregulated and gga-miR-210a-5p was downregulated in LP, and their 121 DE target genes (26 upregulated and 95 downregulated in LCa group) were enriched in calcium signalling pathway, inositol phosphate metabolism, phosphatidylinositol signalling system, pentose phosphate pathways and oxidative phosphorylation. The downregulated genes showed more involvement in these pathways (Fig. 7).

**Figure 8:**
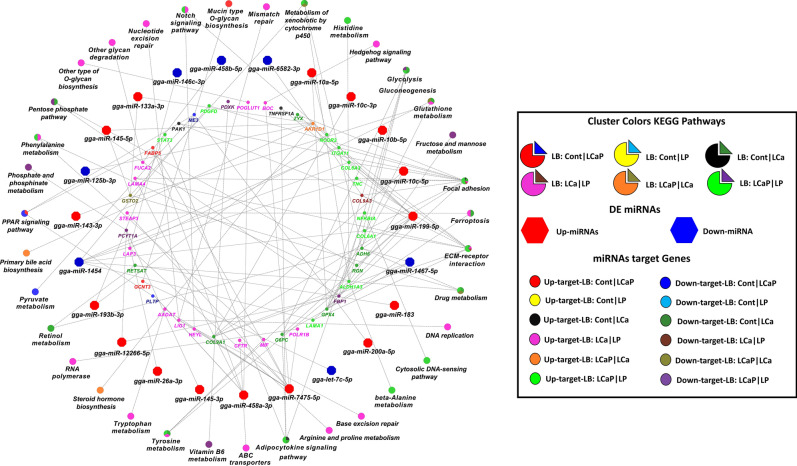
KEGG pathways enrichment analysis of differentially expressed (DE) known miRNAs and their DE target genes from pairwise comparisons between diet groups within LB strain. ClueGO (version 2.5.1) and Cluepedia (version 1.5.7) plugin in Cytoscape (version.3.6.1) environment performed KEGG pathways enrichment analysis. ClueGO generated functionally clustered KEGG annotation network of DE miRNAs and their DE target genes. *P* value was calculated by right-sided hypergeometric tests and Benjamin–Hochberg adjustment was used for multiple test correction. KEGG pathways with a *P* < 0.05 were considered significant.

In the LB strain, 76 DE expressed miRNAs and their 336 DE target genes (192 upregulated and 144 downregulated) were subjected to KEGG pathways enrichment analysis. DE miRNAs and their target genes were significantly enriched in KEGG pathways in Cont *vs* LP, LCaP *vs* LCa and LCa *vs* LP comparisons, whereas no enrichment was observed in Cont *vs* LCaP, Cont *vs* LCa and LCaP *vs* LP comparisons (Fig. 8). In Cont *vs* LP comparison within LB strain, gga-miR-145-3p, gga-miR-199-5p, gga-miR-10c-3p, gga-miR-10c-5p, gga-miR-10a-5p, gga-miR-10b-5p, gga-miR-183 and gga-miR-199-5p were downregulated and gga-miR-1467-5p was upregulated in LP group. These DE miRNAs and their 74 target genes (25 upregulated and 49 downregulated in Cont group) were involved in glycolysis and gluconeogenesis, focal adhesion, ECM receptor interaction, ferroptosis, adipocytokine signalling pathway, pentose phosphate pathway and retinol metabolism. The downregulated genes showed more involvement in these pathways (Fig. 8). In LCaP- *vs* LCa comparison, gga-miR-7475-5p and gga-miR-458a-3p were upregulated and gga-miR-146c-3p was downregulated in LCa group. These 3 DE miRNAs and their 130 DE target genes (97 upregulated and 33 downregulated in LCaP group) were involved in DNA replication, ABC transporters, tyrosine metabolism, focal adhesion, glutathione metabolism, phenylalanine metabolism and notch signalling pathway. The upregulated genes showed more involvement in these pathways (Fig. 8). In LCa *vs* LP comparison, gga-miR-10a-5p, gga-miR-193b-3p, gga-miR-26a-3p, gga-miR-145-3p and gga-miR-12266-5p were downregulated and gga-miR-1454 was upregulated in LCa group. These DE miRNAs and their 61 target genes (37 upregulated and 24 downregulated in LCa group) were involved in focal adhesion, ECM receptor interaction, adipocytokine signalling pathway, drug metabolism, pentose phosphate pathway, glycolysis and gluconeogenesis and notch signalling pathway. The upregulated genes showed more involvement in these pathways (Fig. 8).

### Comparative analysis of KEGG pathway enrichment between LSL and LB strains

**Figure 9:**
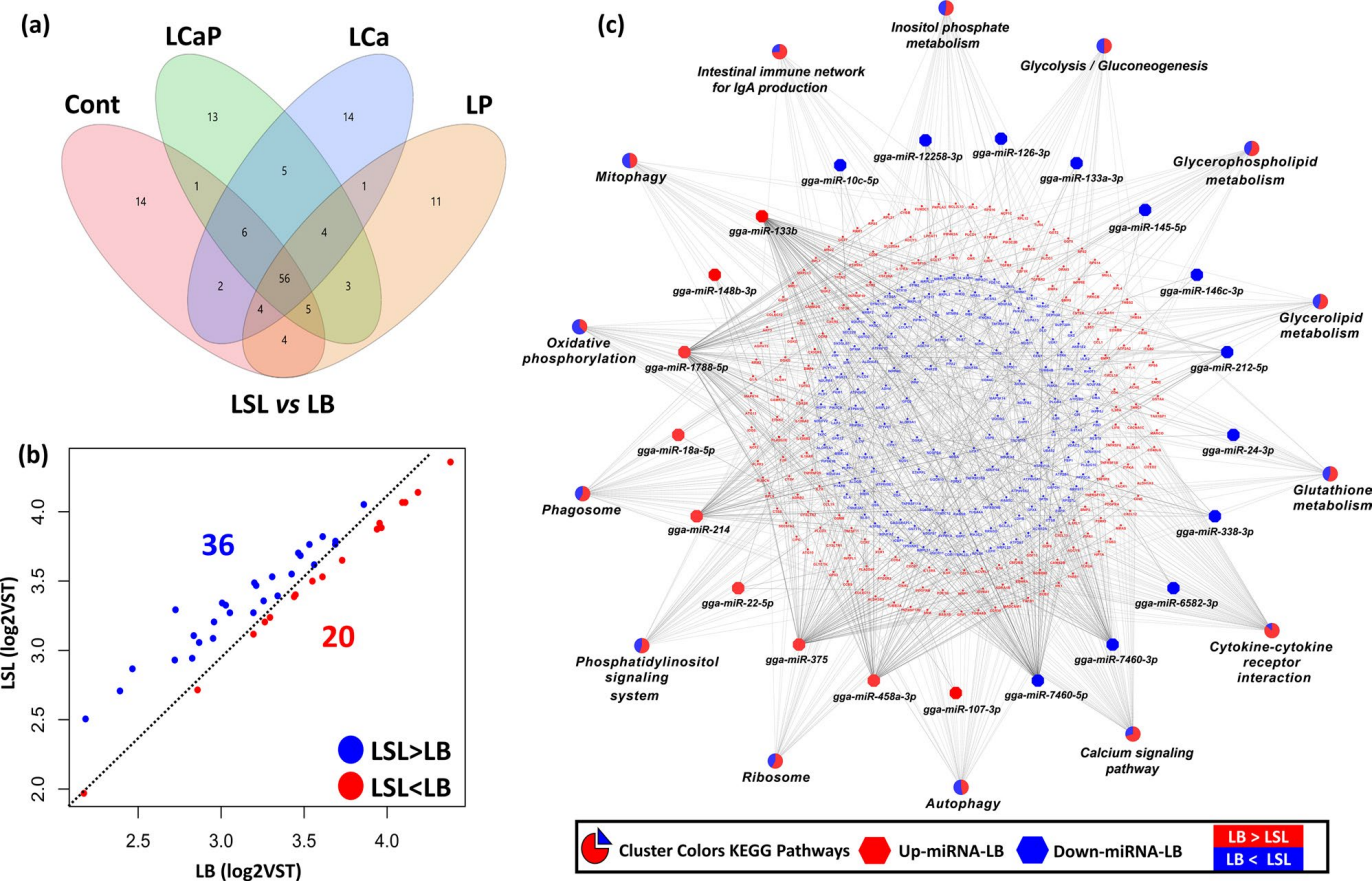
Comparison and KEGG Pathways Enrichment analysis of DE known miRNAs and their DE target genes between LSL and LB strains within diet groups. (a) Venn diagram indicated the deferentially expressed known miRNAs within all four diets groups between LSL and LB strain where 14, 13, 14 and 11 were specific for Cont, LCap, LCa and Lp for both LB and LSL strain respectively and 56 common DE known miRNAs. (b) Scatter plot indicated the deferential expression of 56 common known miRNAs between LSL and LB where 36 miRNAs were downregulated in LB and upregulated in LSL strain and 20 upregulated in LB and downregulated in LSL. (c) ClueGO generated functionally clustered KEGG annotation network of 56 DE known miRNAs and their DE target genes between LSL and LB. *P* value was calculated by right-sided hypergeometric tests and Benjamin–Hochberg adjustment was used for multiple test correction. KEGG pathways with a *P* < 0.05 were considered significant.

Differential expression analysis of known miRNAs between LSL and LB strains within all four diet groups showed 56 common DE miRNAs between both strains independent of diet (Fig. 9a). Out of these 56 miRNAs, 20 miRNAs were upregulated and 36 miRNAs were downregulated in LB strain compared to LSL strain (Fig. 9b). DE target genes of these 56 miRNAs were obtained from the separate study (E-MTAB-9109) and Pearson correlation between 56 DE miRNAs and DE target genes was determined between LSL and LB strains. DE target genes that were negatively correlated with 56 DE miRNAs were considered significant at threshold of 5% FDR. Twenty upregulated miRNAs in LB strain and their 3683 target genes were subjected to ClueGO for KEGG pathway enrichment analysis as cluster one. Similarly, 36 downregulated miRNAs in LB strain and their 2089 target genes were subjected to ClueGO as cluster two. ClueGO generates a functionally annotated KEGG pathway of miRNAs and their downstream targets. We used a less rigorous threshold (*P* < 0.05) and the KEGG pathway which passed the P value threshold was considered significantly enriched. Out of 20 upregulated miRNAs in LB strain, only 9 miRNAs (gga-miR-133b, gga-miR-148b-3p, gga-miR-1788-5p, gga-miR-18a-5p, gga-miR-214, gga-miR-22-5p, gga-miR-375, gga-miR-458a-3p and gga-miR-107-3p) and their target genes showed enrichment. Similarly, out of 36 downregulated miRNAs in LB strain, only 12 miRNAs (gga-miR-10c-5p, gga-miR-12258-3p, gga-miR-126-3p, gga-miR-133a-3p, gga-miR-145-5p, gga-miR-146c-3p, gga-miR-212-5p, gga-miR-24-3p, gga-miR-338-3p, gga-miR-6582-3p, gga-miR-7460-3p and gga-miR-7460-5p) showed enrichment in KEGG pathways. Target genes of 9 upregulated and 12 downregulated miRNAs in LB strain were involved in glycolysis/gluconeogenesis, glutathione metabolism, oxidative phosphorylation, calcium signalling pathway, cytokine-cytokine interaction, ribosomes, phosphatidylinositol signalling system, mitophagy, autophagy, phagosome, glycerolipid metabolism, inositol phosphate metabolism, intestinal immune network for IgA production and glycerophospholipid metabolism (Fig. 9c). Interestingly, the proportion of genes involved in pathways enrichment between LSL and LB strains was different. The genes involved in cytokine-cytokine interaction, calcium signalling pathway, phosphatidylinositol signalling system and intestinal immune network for IgA production were more enriched in LSL strain. Whereas, genes involved in oxidative phosphorylation were more enriched in LB strain.

### Correlation of miRNA and mRNA abundance and phenotypes in relation to P and Ca metabolism

**Figure 10:**
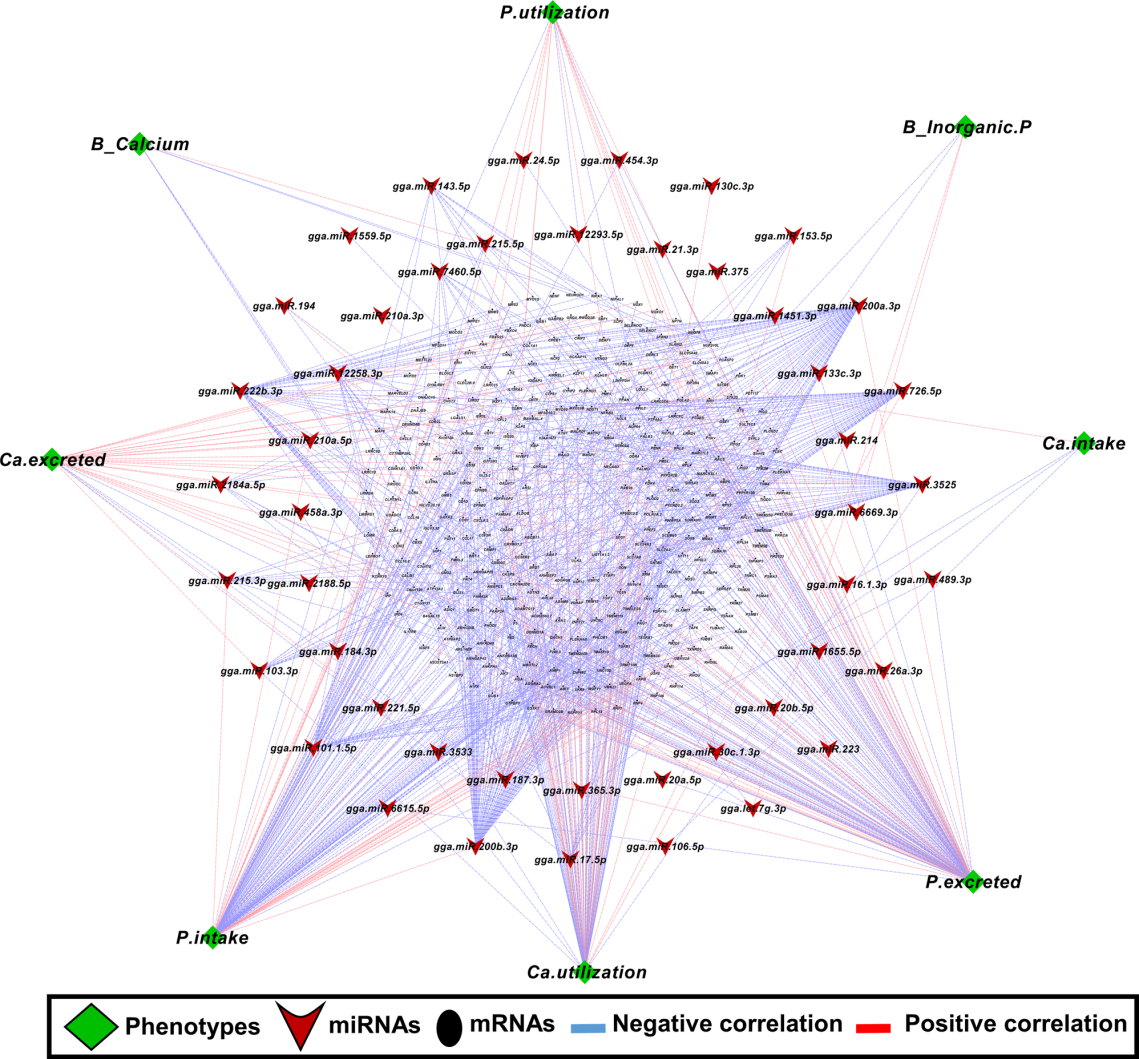
Correlation network analysis of known miRNAs-mRNA from different diet and phenotype of LSL strain. Correlation network was generated using MetScape (version 3.1.3) plugin in Cytoscape (version 3.6.1) environment. The mRNAs with negative correlation with miRNAs were considered significant. Green color diamond shape indicates phenotype data within LSL strain. Red V-shape indicates the deferentially expressed known miRNAs from diet groups within in LSL strain and black ellipse shape indicates the mRNA. Blue line indicates the correlation between Phenotype-miRNA and mRNA.

**Figure 11:**
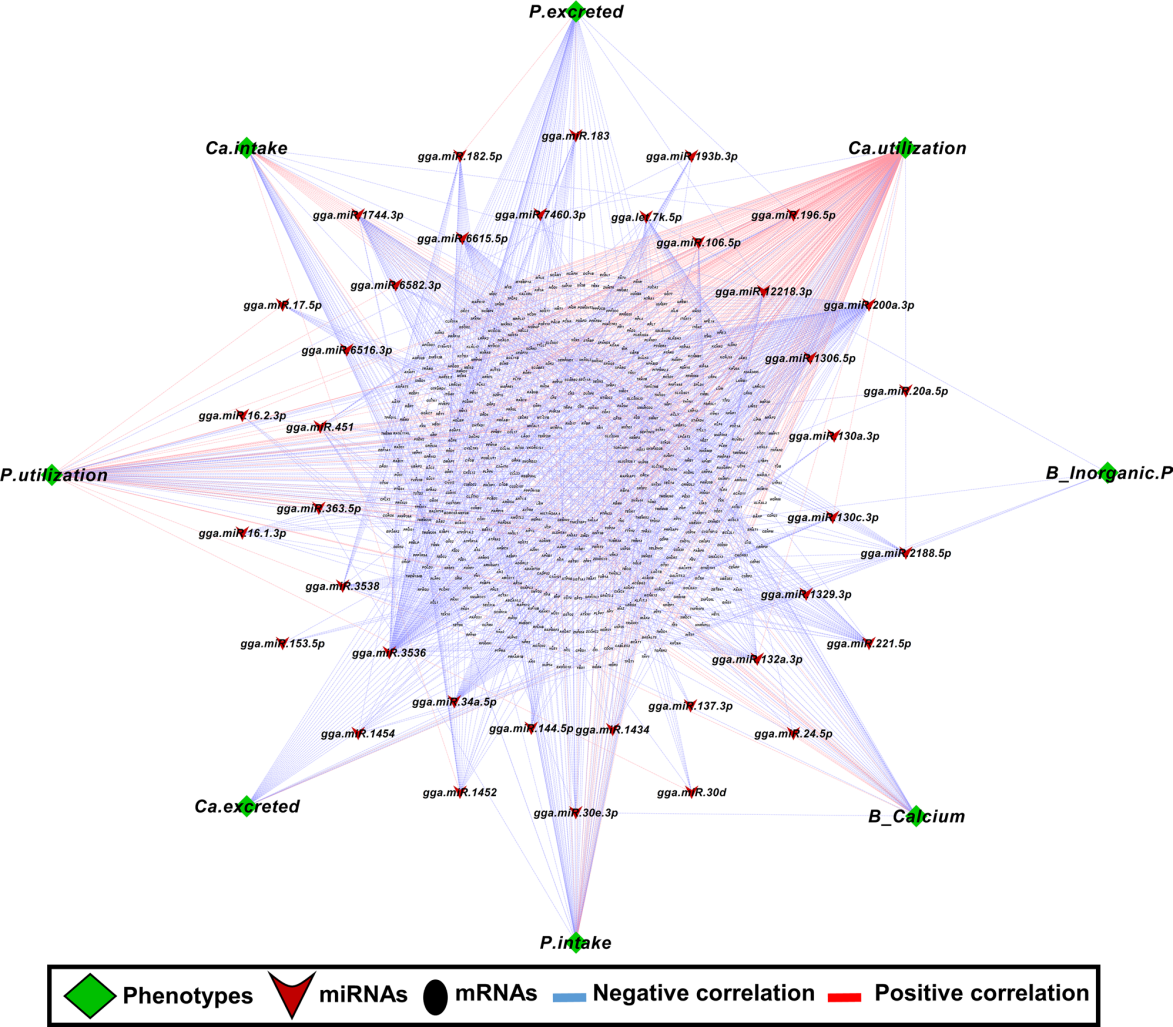
Correlation network analysis of known miRNAs-mRNA and phenotype from different diet groups within LB strain. Correlation network was generated using MetScape (version 3.1.3) plugin in Cytoscape (version 3.6.1) environment. The mRNAs with negative correlation with miRNAs were considered significant. Green color diamond shape indicates phenotype data within LB strain. Red V-shape indicates the deferentially expressed known miRNAs from different diet groups within in LB strain and black ellipse shape indicates mRNA. Blue line indicates the correlation between Phenotype-miRNA and mRNA.

**Figure 12:**
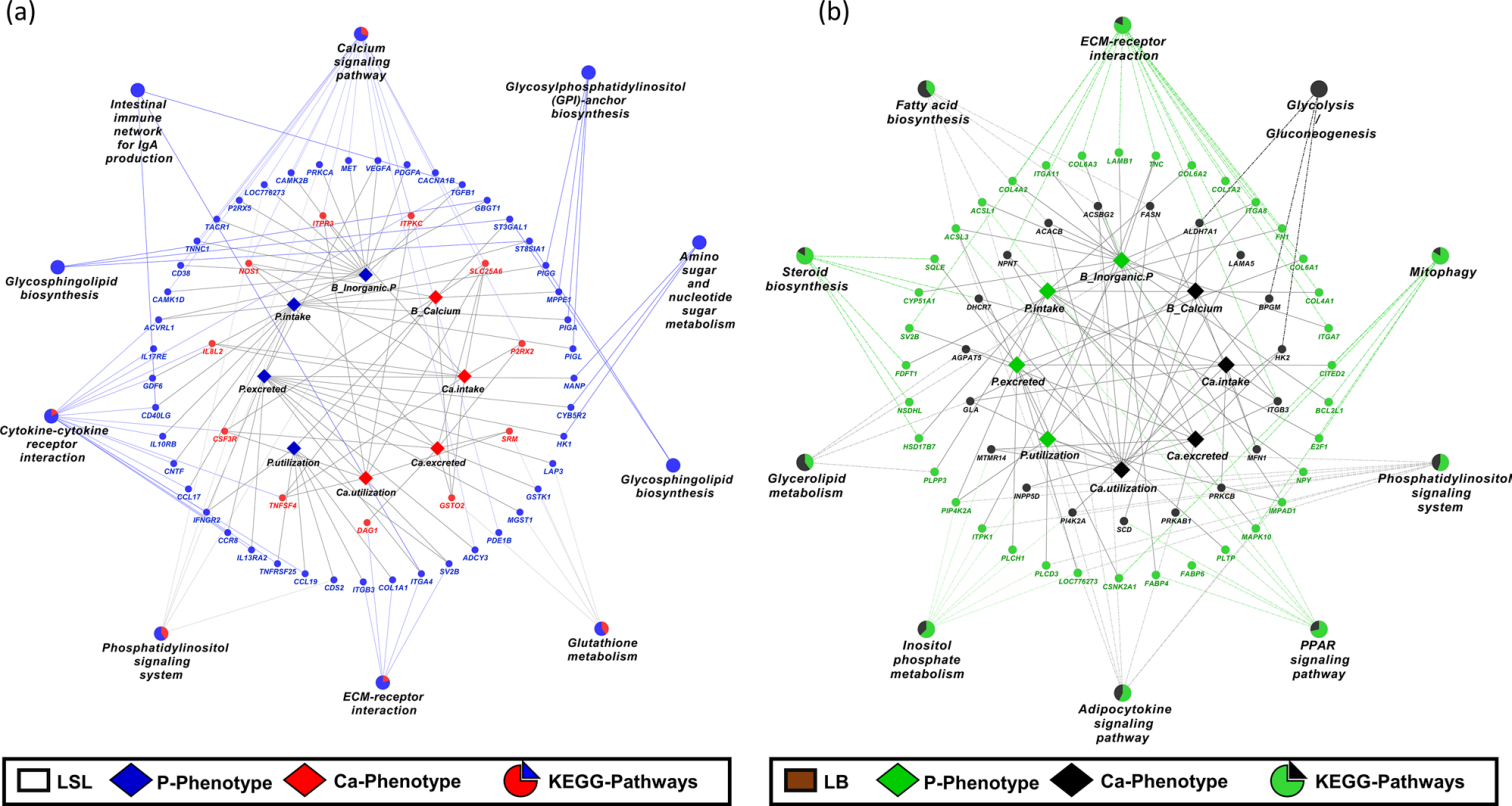
KEGG Pathway analysis of mRNA-phenotype data from different diet groups within LSL and LB strain. (**a**) KEGG pathways enrichment analysis was performed used mRNA which negative correlated with miRNA as showed in Figs. 10 and 11. ClueGO generated functionally clustered KEGG annotation network of correlated phenotype and mRNA data which negative with miRNA from LSL strain. *P* value was calculated by right-sided hypergeometric tests and Benjamin–Hochberg adjustment was used for multiple test correction. KEGG pathways with a *P* < 0.05 were considered significant. Blue ellipse shape indicates the genes which related with phosphorus phenotype and red ellipse shape indicates the genes which related with calcium phenotype. (**b**) Similarly, KEGG pathways enrichment analysis was performed for correlated mRNA-phenotype data which negative correlated with miRNA from LB strain. Green ellipse shape indicates the genes which related with phosphorus phenotypes and black ellipse shape indicates the genes which related with calcium phenotypes.

We used phenotype data related to Ca and P metabolism from our previous study^34^ in order to estimate Pearson correlation coefficients between miRNAs, mRNAs and traits such as Ca intake, P intake, Ca excretion, P excretion, Ca utilization, P utilization as well as inorganic P and Ca levels in blood for LSL and LB strain. A total of 1558 pairs, including 47 miRNAs, 960 mRNAs, 4 Ca-related traits and 4 P-related traits showed significant correlation with each other (*P* < 0.05) in LSL strain (Fig. 10). In the LB strain, 1985 pairs including 38 miRNA,1092 mRNA, 4 Ca-related traits and 4 P- related traits were significantly correlated with each other (*P* < 0.05) (Fig. 11). We classified the phenotypes into two categories, the first category related to calcium metabolism and the second category related to phosphorus metabolism, for KEGG pathway enrichment analysis (Fig. 12a,b). In the LSL strain, P metabolism-associated transcripts were mainly enriched in the intestinal immune network for IgA production, glycosphingolipid biosynthesis, and amino sugar and nucleotide sugar metabolism, whereas Ca metabolism-associated transcripts were proportion enriched in the calcium signaling pathway, glutathione metabolism, and the phosphatidylinositol signaling system. In the LB strain, both P- and Ca-related phenotypes associated transcripts were involved in steroid biosynthesis, inositol phosphate metabolism, and the adipocytokine signaling pathway. Transcripts of glycolysis/gluconeogenesis were enriched only in Ca-related traits.

### Validation of NGS miRNA data and qPCR miRNA data

**Figure 13:**
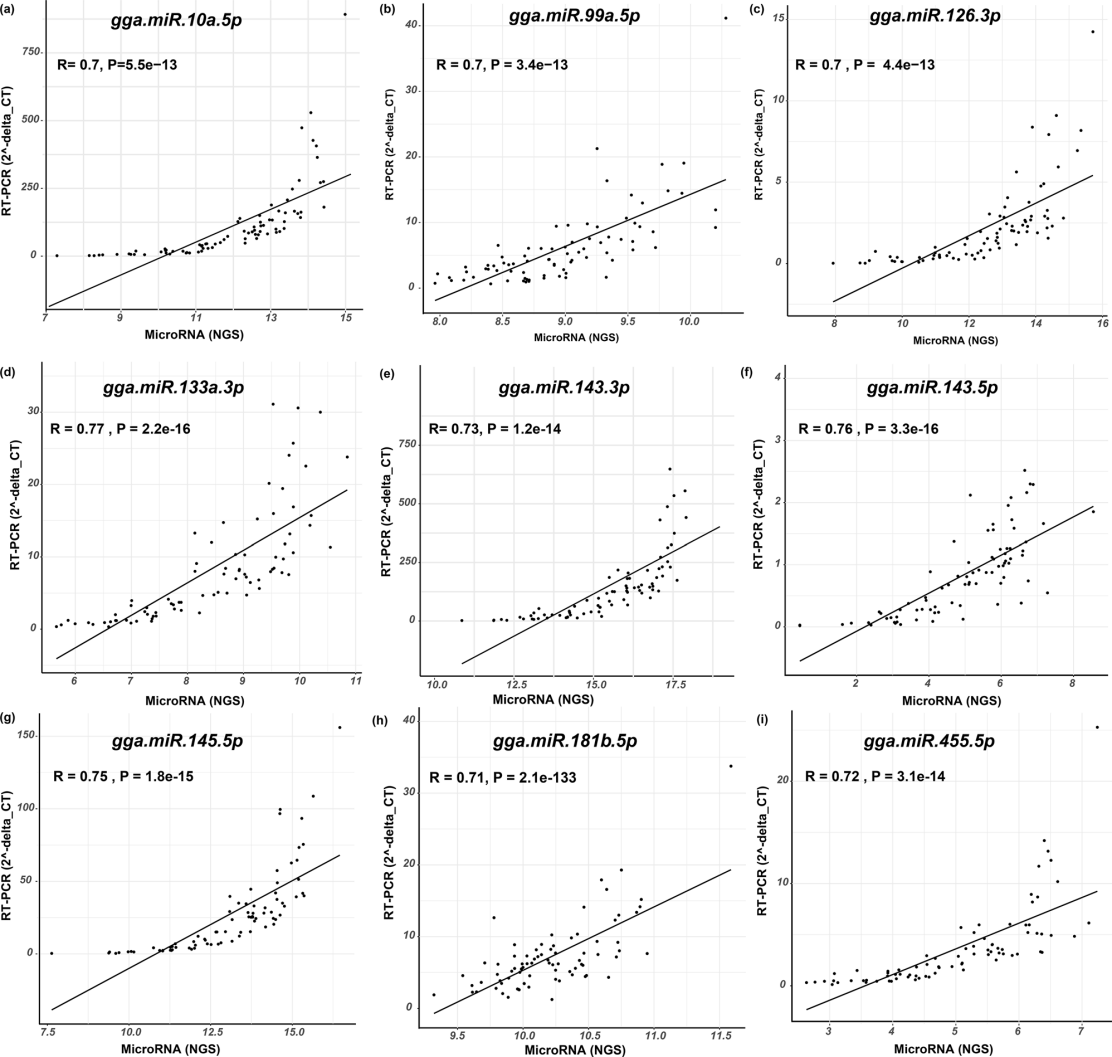
Validation of qPCR and NGS data of selected miRNAs. Scatter plots indicated the validation of NGS selected miRNAs data and qPCR miRNA with respect to their coefficient of correlation (r) ranging from 0.70 to 0.76 and *P* < 0.05.

Finally nine miRNAs (gga-miR-10a-5p, gga-miR-133a-3p, gga-miR-143-3p, gga-miR-143-5p, gga-miR-99a-5p, gga-miR-145-5p, gga-miR-181b-5p, gga-miR-455-5p and miR_126_3p) were validated by qPCR as shown in Fig. 13. Our NGS miRNA data and qPCR miRNA data of the identical samples (n = 80) showed good consistency with the coefficient of correlation (r) ranging from 0.70 to 0.76 and P < 0.05 for all validated genes.

## Discussion

Calcium is highly abundant in the egg shell, but adequate amount of P is critical for proper utilization of Ca. Both Ca and P are important components of poultry diet and an inadequate Ca and P supply can deteriorate both egg quality and health of the bird. Absorption and utilization of Ca and P in laying hens is also affected by their interaction with other nutrients, genetic strain of the hens, and environmental conditions^13^. In this study we investigated the effect of Ca and P quantity in diets and the effect of strain on miRNAs expression in jejunum of LSL and LB laying hen strains. The aim of this study was to identify miRNAs and their downstream pathways that can be potentially involved in P metabolism, absorption or utilization and other metabolic processes. Our data shows that changing the quantity of Ca or P in poultry diet affects changes in the expression of miRNAs involved in different biological pathways.

When comparing Cont *vs* LCaP groups in LSL strain, gga-miR-10c-5p, gga-miR-129-5p and gga-miR-1663-3p were downregulated and gga-miR-193b-3p and gga-miR-455-5p were upregulated in LCaP group. The gene ontology analysis of these miRNAs and their target genes showed the involvement of these miRNAs in nucleoside phosphate metabolic process. The miRNA gga-miR-1663-3p also showed an association with glutathione metabolism by targeting Glutathione Peroxidase 1 (*GPX1*) (Fig. 7). Similarly, in Cont *vs* LP comparison, gga-miR-12243-5p is downregulated in the LP group. MiR-12243-5p regulates glutathione metabolism by targeting Glutathione Peroxidase 4 (*GPX4*) and Immunoglobulin Binding Protein 1 (*IGBP1*) (Fig. 7). Glutathione acts as an antioxidant and also plays a significant role in nutrient metabolism and signal transduction^39^. Results suggest a connection between the glutathione signaling pathway and Ca and especially P, which is mediated by miRNAs. Moreover, in Cont *vs* LP comparison, gga-miR-129-5p was downregulated in LP group. MiR-129-5p regulates oxidative phosphorylation by targeting NADH:Ubiquinone Oxidoreductase Subunit A6 (*NDUFA6*), Ubiquinol-Cytochrome C Reductase Complex III Subunit VII (*UQCRQ*) and Inorganic Pyrophosphatase 2 (*PPA2*). MiRNAs gga-miR-12243-5p and gga-miR-129-5p also regulate inorganic ion homeostasis including P and Ca. Other miRNAs differentially expressed in Cont *vs* LP comparison are involved in mitochondrial dysfunction and immune related pathways. In LCa *vs* LP comparison, gga-miR-30e-3p was downregulated and gga-miR-210a-5p, gga-miR-204 and gga-miR-191-3p were upregulated in LP group. These miRNAs are involved in ribose phosphate metabolism, developmental processes and mitochondrial dysfunction (Figs. 5b, 7). Additionally, gga-miR-210a-5p is also involved in inositol phosphate metabolism by targeting Phospholipase C Gamma 1 (PLCG1) (Fig. 7). PLGC1 protein catalyses the formation of inositol 1, 4, 5—triphosphate from phosphatidylinositol 4, 5-bisphosphate, a reaction which uses Ca as a co-factor^40^.

Next, we analysed the effect of different Ca and P dietary concentrations on jejunum mucosa miRNAs expression in the LB strain. In Cont *vs* LP comparison, gga-miR-199-5p was downregulated in the LP group, and KEGG pathway analysis shows that gga-miR-199-5p is involved in pentose phosphate pathway by targeting Regucalcin (*RGN*) (Fig. 8). Other differentially expressed miRNAs in this comparison are involved in cofactor metabolic processes, organic anion transport, carbohydrate metabolism, regulation of hormone levels and glycerophospholipid metabolism (Fig. 6b). In LCaP *vs* LCa comparison, gga-miR-7475-5p, gga-miR-1788-5p, gga-miR-726-5p and gga-miR-6582-3p were downregulated, and gga-miR-458-3p was upregulated in LCa group. According to KEGG pathway and gene ontology analysis, most important function of these miRNAs is regulation of ion transport, including P and Ca transport, by targeting different genes. Other than ion transport, these miRNAs are also involved in muscle and organ development and regulate ABC transporters (Figs. 6b, 8). In LCa *vs* LP, gga-miR-10a-5p and gga-miR-193b-3p were upregulated in LP group. These miRNAs regulate glutathione metabolism by targeting Nuclear Receptor Corepressor 2 (*NCOR2*). NCOR2 protein is involved in transcription silencing of certain target genes. MiRNA gga-miR-12266-5p was also upregulated in LP group and it regulates pentose phosphate pathway by targeting Fructose Bisphosphatase 1 (*FBP1*). Additionally, gga-miR-26a-3p, gga-miR-145-3p and gga-miR-12266-5p were upregulated in LP group, which regulate phosphinate and phosphonate metabolism by targeting Phosphate Cytidylyltransferase 1 Alpha (*PCYT1A*). PCYT1A protein is involved in phosphatidylcholine biosynthesis.

When analysing LSL and LB strains side by side, our data revealed 56 differentially expressed miRNAs between this two strains commonly observed in all diet groups (Fig. 9a). Previous studies reported transcript different among LSL and LB and enriched in immune system processes and phosphorus metabolic processes^36,37^. We added another level of transcript regulation by using miRNA in this study and found respective target genes enriched in interesting KEGG pathways such as glycolysis/gluconeogenesis, glutathione metabolism, oxidative phosphorylation, calcium signaling pathway, cytokine-cytokine interaction, ribosomes, phosphatidylinositol signaling system, glycerolipid metabolism, inositol phosphate metabolism, intestinal immune network for IgA production and glycerophospholipid metabolism (Fig. 9c). Interestingly, the target genes involved in cytokine-cytokine interaction, calcium signalling pathway, phosphatidylinositol signalling system and intestinal immune network for IgA production were more enriched in LSL strain, whereas, genes involved in oxidative phosphorylation were more enriched in LB strain (Fig. 9c). MiRNA gga-miR-148b-3p downregulated in LSL is well-known miRNA to be associated with osteogenic differentiation^41^, and involved in bone remodeling^42^. In addition miR-148b-3p was identified as molecular marker in the gut of quail which represent extremes for P utilization^33^. Recently, it was reported that gga-miR-146c upregulation occurs upon Mycoplasma gallisepticum and the miRNA significantly increased the expression of TLR6, NF-κB p65, MyD88, and TNF-α^43^. This finding is consistent with our result that gga-miR-146c-3p is upregulated in LSL and enriched in immune system processes. Interestingly, we found that miRNAs such as gga-miR-7460-5p, gga-miR-7460-3p, gga-miR-24-3p, gga-miR-212-5p, gga-miR-146c-3p, gga-miR-133b, gga-miR-1788-5p, gga-miR-458a-3p, gga-miR-375 and their target genes were prominently involved in inositol phosphate metabolism and phosphatidylinositol signaling. Our findings indicate that the aforementioned miRNAs are important regulators in P signaling and inositol phosphate metabolism (Fig. 9c).

As outlined in our previous study using the same samples, there were no significant differences among the strains P- and Ca-related traits, including intake, excretion, and utilization of P and Ca, respectively^34^. In the present study the correlation analyses of miRNAs and target mRNAs, with the phenotypes revealed a strain-specific fine-tuning of molecular routes depending on P- and Ca-supplies. In particular, both P- and Ca- related phenotypes in the LSL strain were enriched in immune, glutathione metabolism or calcium signaling pathways, but in metabolic signaling in the LB strain. This fine-tuning at the molecular level reflect different physiological strategies in Ca-P homeostasis. The results suggest that genetic background plays an important role in Ca-P homeostasis through miRNA regulation.

Several miRNAs described in this study regulate important metabolic and developmental processes, and mineral homeostasis. We speculate that some of these miRNAs can be secreted in jejunum lumen and interact with intestinal epithelium and gut microbiota. We further suggest that these miRNAs might be linked with microbial phytase synthesis. This study provides a basis for exploring miRNA networks in the intestinal mucosa and understanding their role in P absorption and utilization. The results obtained, and in particular, the finding of different miRNA-directed molecular routes used to maintain mineral homeostasis under variable P and Ca supply in the two strains, serve as a basis for further studies on the role of miRNAs in maintaining mineral homeostasis in laying hens.

## Methods

### Experimental design

This study was part of this experiment which was designed on the basis of a 2 × 2 × 2-factorial arrangement of treatments Sommerfeld et al.^35^. In brief, four dietary combinations with varying Ca levels (recommended *vs.* 15% reduction) and P levels (recommended *vs.* 20% reduction) for each strain were used. Previously documented concentrations of Ca and P were used as control^44^. Reduced concentration of Ca and P in feed were obtained by reducing the quantity of monocalucium phosphate and/or limestone. The control diet contained (Cont) 39.5 g Ca/kg dry matter (DM) and 5.3 g/kg (DM) respectively. The other dietary groups were supplied with low levels of both Ca and P (LCaP) at 34.0 g/kg DM and 4.7 g/kg DM respectively, or low Ca but standard P level (LCa) at 35.1 g/kg DM and 5.3 g/kg DM respectively, or low P but standard Ca level (LP) at 40.3 g/kg DM and 4.7 g/kg DM respectively. The feed ingredients did not contain plant phytase and exogenous phytase was not added to the feed. There were 8 experimental groups in this study (4 dietary groups for LB and 4 dietary groups for LSL) and each dietary group contained 10 birds. For this purpose, 10 roosters per strain were chosen and four randomly chosen offspring of each rooster were chosen and assigned to one of the dietary treatments. In first 26 weeks of life, the birds were kept together in floor pens. Afterward, birds were rehoused separately into metabolism coalition cages and were phenotyped for zoo-technical and physiological characteristics. Birds were slaughtered at the age of 31 weeks, after stunning by gas. For each bird, a 2 cm long jejunum sample was collected, nearly 3 cm distal to the duodenal loop, and stored at – 80 °C.

### RNA extraction, miRNA library preparation and sequencing

TRIzol Reagent (Invitrogen, Karlsruche, Germany) was used to extract the total RNA from all of the 80 jejunum samples. Small RNA (miRNA) fractions were obtained by using the RNeaasy Mini kit (Qiagen) and consequently miRNeasy Mini kit (Qiagen). Agilent RNA 600 Nano kit and 2100 Bioanalyzer system (Agilent Technologies) were used to examine the reliability of total RNA. The quantity and quality of RNA was determined using NanoDrop ND-2000 (Peqlab, Erlangen, Germany) and Bioanalyzer 2100 devices (Agilent Technologies, Waldbronn, Germany). RNA integrity numbers were between 7.1 and 9.4. Small RNA sequencing libraries were generated from 1 µg of enriched small RNA using the NEXTFLEX Small RNA-Seq Kit v3 for Illumina according to the manufracturer's recommendations (Bioo Scientific Corp). The smRNA-seq libraries were quality assessed for expected fragment length of a major peak at about 152 bp using an Agilent High Sensitivity DNA kit and 2100 Bioanalyzer (Agilent Technology) and then size-selected using the BluePippin System and 3% agarose gel cassette with the internal Q2 DNA marker (Sage Science). The molar concentration of the libraries was determined using a Qubit dsDNA HS assay kit (Invitrogen) and normalized to 2 nM prior to sequencing. Next, the sequencing of reads was performed at the Institute of Genome Biology, FBN Dummerstorf Germany by using the HiSeq2500 sequencing platform to sequenced 50 bp single-end-reads. The base call (BCL) format files were attained from the sequencer and then converted into FASTQ format files by using bcl2fastq2 conversion software. v2.19. The quality controls of raw fastq files were checked via FASTQC, V.011.^45^. The raw data were deposited to the public repository under ArrayExpress accession number E-MTAB-9136.

### Pre-processing: adapter trimming, quality control and read mapping

Earlier, Small RNA library raw reads were gathered from 80 jejunum samples by using illumina HiSeq Sequencer. The raw-fastq reads were pre-processed according to the user guide instruction (NEXTFLEX Small RNA-Seq Kit v3 for Illumina) including trimming of low-quality base, adapter-like sequence and the first and last 4 bases of adapter-clipped reads using Trim Galore version 0.6.5 (https://github.com/FelixKrueger/TrimGalore) and FastQC tools^45,46^. Afterward, the contaminated reads were filtered out which was not according to cut-off criteria. The reads which were 18 to 33 nucleotide long in length and the quality Phred Score ≥ 20 were considered for further analysis. Next, the reads that passed all filtering criteria were used for mapping. We have overall 80 fastq files for both LB and LSL strains and for each strain we have 40 fastq files which were mapped to Gallus gallus reference genome (GRCg6a) from ensemble by using bowtie v1.2.2 with default parameters^47,48^.

### Identification of known and novel miRNA by miRDeep2

The novel and known miRNAs were identified and quantified by using miRDeep2 v2.0.08 (https://github.com/rajewsky-lab/mirdeep2)^49^. *Gallus gallus* genome was used as reference, and the genomes of *Homo sapiens* and *Mus musculus* were considered as related species. To avoid redundancy of miRNA identifiers in the output read count matrix from miRDeep2, mature miRNA sequences were used. To further resolve remaining duplications of miRNA identifiers, we considered only the maximum number of reads for each mature miRNA. Subsequently, a threshold of 80% quantile of log counts per million (log CPM) of each experimental group was applied to discard low expression miRNAs. After excluding the low expression miRNAs, 576 miRNAs were shortlisted which were used for further downstream analysis.

### Remove batch effects and differential expression analysis

After performing filtration steps, the data was not clean and still showed some batch effects. To reduce the unwanted variation in miRNA sequence and enhanced data quality, the RUVSeq R package was used^50,51^. We used the RUVr function of RUVSeq for assessment of batch variables (BVs) according to the guidelines provided in RUVSeq vignette (package version: 1.18.0). The RUVr function played a vital role in the calculation of residuals from the model fitted with all preferred outcomes, in our case strain, diet, and sequencing lane. In the final model, we included the first two batch variables (BV1 and BV2), after the assessment of all possible batch variables. Later on, the differential expression analysis was performed using DESeq2 R Package function DESeq() with default parameters^52^. The ultimate model used for differential expression analysis was [strain, diet, sequencing lane, and batch variables (BV1 and BV2)].

### Prediction of downstream miRNA targets and their correlation network analysis

To investigate the downstream target mRNAs for differentially expressed (DE) miRNAs, we extracted 5106-3-UTR sequences, 5662-5-UTR sequences and 15732 coding sequences from most recent genome assembly (GRCg6a) of *Gallus gallus* based on Ensembl annotation version 97. These sequences were fragmented into 2-kilobase (2 kb) fragments with a 50 bases overlap. RNAhybrid version 2.1.2 was used to predict the potential miRNA-targets by setting the parameter as binding energy cut-off 25 k, helix constraint ranges from 2 to 7 and one hit per target^53^.

MiRNAs and their downstream mRNA targets were selected based on their minimum free energy and p-value. Initially, the count matrices of miRNA and mRNA were transformed to variance-stabilizing transformation format by using DESeq2. The mRNA count matrices of jejunum samples were obtained from the same animals in a corresponding study with accession number E-MTAB-9109. The Pearson correlation between miRNA and mRNA was calculated for all dietary combinations of LSL and LB strains. The correlation-based network was generated by using the MetScape (version 3.1.3) plugin in the Cytoscape (version 3.6.1) environment^54^.

### Enrichment analysis of miRNAs and target mRNAs

Differentially expressed miRNAs and their downstream differentially expressed mRNA targets were selected from correlation networks. The mRNAs negatively correlated with miRNAs were used for downstream gene ontology analysis using Metascape (gene annotation and analysis resource). All genes were converted to the human gene symbols by using orthologue conversion method in Metascape^55^. All statistically enriched biological processes were identified on the basis of accumulative hyper-geometric P values, and the enrichment factor was used to filter most significantly enriched biological processes. After filtration, remaining significant terms were hierarchically clustered into a tree, based on Kappa-statistical similarities among their genes. A Kappa-score of 0.3 was applied to cast the tree into top-level biological term clusters. The term within each cluster were exported as excel spread sheet and selected the top five child biological terms which showed enrichment in our subjected gene list of different dietary groups according to their p-values. GO circular plots were generated by using Goplot v.1.0.2 package within R programming environment^56^. KEGG pathways analysis was done by using ClueGO (version 2.5.1) and Cluepedia (version 1.5.7) plugin in Cytoscape (version.3.6.1) environment^57–59^. The parameters used for ClueGO analysis were a hypergeometric test that was used for enrichment analysis and Benjamini–Hochberg correction was used for multiple testing correction and the *Gallus gallus* genome assembly as a reference. ClueGO generates a functionally annotated KEGG pathway of miRNAs and their downstream targets. The KEGG pathway, which passed the P value threshold (*P* < 0.05), was considered significantly enriched.

### Validation of miRNA NGS data by real-time RT-PCR

Individual small RNA samples from all groups were used for qPCR using the Fluidigm BioMark HD System to validate miRNA NGS data. Briefly, 250 ng of each small RNA sample was reverse transcribed using reverse transcription kit (Invitrogen) following the manufacturer's protocol. For specific target amplification, 5 μL pre-amplification sample mixture for each cDNA was prepared by mixing 2.5 μL PreAmp Master Mix, 1.25 μL of cDNA, 1 μL PreAmp Master Mix, 0.5 μL Pooled Delta Gene Assay Mix (500 nM) and 0.75 μL water. These reactions were incubated at 95 °C for 10 min, followed by 10 cycles of 15 s at 95 °C and 4 min at 60 °C, and then infinite hold at 4 °C. After incubation, the samples were cleaned using exonuclease I treatment method. Cleaned samples were diluted tenfold using DNA suspension buffer. Fluidigm quantitative measurement runs were carried out with 96.96 dynamic arrays (Fluidigm Corporation, CA, USA) according to manufactures instructions. The data were analyzed with real-time PCR analysis software in the BioMark HD instrument (Fluidigm Corporation, San Francisco, CA). 18S and SNORD21 were used as housekeeping controls. The correlation of the results between the two techniques was assessed using correlation coefficients of Pearson. The miRNA primers sequence was showed in supplementary (Table S1).

### Supplementary materials

The sncRNAseq data that support the findings of this study are available in ArrayExpress with the accession number (E-MTAB-9136).

### Ethical statement

The study was performed at the Agricultural Experiment Station of the University of Hohenheim, Germany with strict compliance with the German Animal Welfare Legislation. Animal experimentation was approved by the Regierungspräsidium Tübingen, Germany (Project no: HOH50/17TE). All experiments were performed in accordance with relevant guidelines and regulations of the German Law of Animal Protection. The study was also carried out in compliance with the ARRIVE guidelines.

### Ethics approval and consent to participate

The study was approved by the Regierungspräsidium Tübingen, Germany (Project No. HOH50/17TE) in accordance with the German Animal Welfare Legislation.

## Supplementary Information

Supplementary Information.
